# Recent Developments in Mycobacteriology: A Clinical and Diagnostic Perspective

**DOI:** 10.1155/2014/813185

**Published:** 2014-04-30

**Authors:** Tomasz Jagielski, Jakko van Ingen, Nalin Rastogi, Jarosław Dziadek

**Affiliations:** ^1^Department of Applied Microbiology, Institute of Microbiology, Faculty of Biology, University of Warsaw, I. Miecznikowa 1, 02-096 Warsaw, Poland; ^2^Department of Medical Microbiology, Radboud University Medical Centre, P.O. Box 9101, 6500 HB Nijmegen, The Netherlands; ^3^WHO Supranational TB Reference Laboratory, TB and Mycobacteria Unit, Institut Pasteur de la Guadeloupe, 97183 Abymes, France; ^4^Mycobacterium Genetics and Physiology Unit, Institute of Medical Biology, Polish Academy of Sciences, Lodowa 106, 93-232 Łódź, Poland

Mycobacteria comprise a diverse group of bacteria that are widely distributed in nature, some of which cause significant diseases in humans. The most prominent representative of this group is the* Mycobacterium tuberculosis *complex, which includes the etiological agents of tuberculosis (TB), which, with over 8 million new cases and nearly 2 million deaths annually, continues to cause one of the major health burdens for humans. This complex also includes the causative agents of TB in animals, of which* Mycobacterium bovis*, the causative agent of bovine TB, is the most prominent. Whereas the prevalence of TB has been closely monitored in most parts of the world, the epidemiology of nontuberculous mycobacterial (NTM) infections remains poorly defined, in spite of the fact that the importance of NTMs as a cause of opportunistic infections of humans has been increasingly recognized over the last two decades, particularly in areas where the incidence of TB is in decline.

The purpose of this special issue is to provide the reader with some recent achievements in mycobacteriology, with particular emphasis on the developments which have direct relevance to the clinical practice and diagnostic performance.

The issue opens with a comprehensive state-of-the-art review on the molecular typing methods for* M. tuberculosis* and some NTM species, most commonly associated with human disease. For the various methods, technical practicalities as well as discriminatory power and accomplishments are discussed.

The next two studies focus further on genotyping methods in the molecular epidemiology of TB. Both describe the principle, technical advantages, and discriminatory power of the ligation-mediated PCR systems, namely, fast ligation amplification polymorphism (FLAP) method and fast ligation-mediated PCR (FLiP) (A. Żaczek et al.).

Genotyping methods allow for delineating phylogenetic relationships between the strains. To improve the utility of genotyping and to increase international consensus in the interpretation of genotyping results, M. Aminian et al. propose a new algorithm, publicly available online, useful for classification of* M. tuberculosis* complex isolates into phylogenetic clades, based on the spoligotyping profiles.

Molecular interrogation has entered both the epidemiological and the clinical field today. For clinical practice, molecular tools can help in detecting mycobacteria in clinical samples as well as detecting markers for drug resistance. In this context, Z. Bakuła et al. examine the mutational “hot spots” in the* embB* gene potentially related with resistance of* M. tuberculosis* to ethambutol (EMB). Similarly, but from a different angle, N. Alvarez et al. investigate the molecular mechanisms behind resistance of tubercle bacilli to fluoroquinolones and the binding of levofloxacin to the mycobacterial DNA gyrase in particular.

Identification of* M. tuberculosis* complex isolates by nonmolecular means is described in the contribution by D. Machado et al. Here, the usefulness of the MGIT TBc Identification Test (Becton Dickinson), an immunochromatographic assay which detects the MPB64 protein, is evaluated for routine identification of* M. tuberculosis* complex in a network of hospital mycobacteriology laboratories in Portugal and other Portuguese-speaking countries.

Despite all advances in clinical diagnostics and molecular epidemiology, there still is no truly effective vaccine against TB. In a broad stream of research on new anti-TB vaccines is a study of M. V. Bianco et al., who investigate the efficacy of a new vaccine candidate against TB based on a* M. bovis* mutant in* p27–p55* operon.

Pharmacokinetic analyses are gaining foothold in the treatment of TB, where they are available. An interesting issue is addressed by A. Zabost et al., who explore the correlation between the N-acetyltransferase 2 (NAT2) genotype with isoniazid (INH) acetylation in TB patients and demonstrate NAT2 genotyping as an important tool for the adjustment of INH dosing regimens.

Four studies deal with NTM. The first one focuses on* Mycobacterium kansasii* which is among the most frequently isolated NTM species from human clinical cases worldwide. Since only certain genotypes of* M. kansasii* are clinically important, a paper by Z. Bakuła et al. gives a snapshot of the distribution of* M. kansasii* subtypes among patients in Poland suspected of having pulmonary NTM disease. In the second paper, M. Slany et al. report on a distinct NTM skin disease in humans, known as fish tank granuloma and caused by* Mycobacterium marinum*. The remaining two NTM papers come from the veterinary field. C. Goepfert et al. report cases of* Mycobacterium avium* subsp.* avium* infection in veal calves, highlighting the diagnostic problems surrounding these infections. A. Ledwoń et al. report the results of the experimental infection with* M. avium* subsp.* avium* in budgerigars positive for the beak and feather disease circovirus (BFDV) in order to determine how mycobacteriosis affects the course of the viral disease.

The articles within this issue differ considerably from each other with respect to their research scopes and methodologies, thus reflecting the multidirectional character of the research in the ever-expanding field of mycobacteriology. We face an exciting era in mycobacteriology, where greater understanding of the mycobacteria leads to technical improvements that change clinical practice and these techniques in turn help to curb the epidemic of mycobacterial diseases of humans and animals.

*Tomasz Jagielski* *Jakko van Ingen* *Nalin Rastogi* *Jarosław Dziadek*

